# When care meets resistance: professional nurses’ real-world struggles in providing primary health services to LGBTQI communities in Amahlathi, Eastern Cape Province, South Africa

**DOI:** 10.3389/fmed.2026.1763881

**Published:** 2026-03-25

**Authors:** M. Dube, L. E. Manamela, G. O. Sumbane, L. W. Mokhwelepa

**Affiliations:** 1 Nursing Science Department, Faculty of Health Science, School of Health Care Science, University of Limpopo, Polokwane, South Africa; 2 Faculty of Health Science, School of Medicine, University of Limpopo, Polokwane, South Africa

**Keywords:** challenges, LGBTQI, primary health care, professional nurses, struggles

## Abstract

**Background:**

Access to equitable primary healthcare (PHC) for LGBTQI individuals remains a persistent challenge worldwide, yet little is known about how professional nurses (PNs) in rural South African contexts experience and navigate these challenges.

**Objective:**

The study aimed to explore and describe the lived experiences of professional nurses in providing PHC services to LGBTQI patients in Amahlathi Municipality, Eastern Cape Province, South Africa.

**Methods:**

For this research, a descriptive phenomenological research design was used to obtain rich, in-depth accounts of the real-world experiences of nurses. A non-probability purposive sampling method was used to select 23 professional nurses working in PHC facilities in the Amahlathi Sub-district, Eastern Cape. Data were collected through semi-structured face-to-face interviews, which were then analyzed using Colaizzi’s phenomenological data analysis method. Measures to ensure trustworthiness and ethical considerations were maintained throughout the study.

**Results:**

The findings showed that professional nurses face significant competency gaps, personal biases, and confusion about LGBTQI identities, which negatively affect the delivery of services. They also revealed that LGBTQI patients often fear discrimination, withhold sensitive information, and display defensive behaviors due to past negative experiences. Consequently, these combined provider- and patient-related challenges strain clinical interactions and hinder equitable primary healthcare access in Amahlathi.

**Conclusion:**

The study revealed that professional nurses in the rural Eastern Cape face both personal and systemic obstacles to providing inclusive care to LGBTQI individuals. Enhancing nurses’ competencies through targeted LGBTQI-focused training, creating supportive clinical environments, and strengthening policy frameworks are critical to improving the quality of care.

## Introduction

Globally, LGBTQI communities continue to face serious barriers within mainstream healthcare systems, despite growing recognition of the need for inclusive and rights-based care (1). Studies from various high-income countries demonstrate that professional nurses (PNs) often lack essential competence in managing the unique health needs of gender-diverse patients, resulting in clinical mismanagement, stigma, and care avoidance (2–4). Furthermore, existing evidence reveals consistent patterns of judgmental attitudes, misgendering, and insensitivity, which undermine patient trust and compromise health outcomes (5, 6). Similarly, confusion about gender identity terminology and fear of offending often lead nurses to avoid meaningful engagement with LGBTQI patients, consequently creating unmet health needs (7, 8).

In sub-Saharan Africa, these challenges are further intensified by cultural norms and legal frameworks that often reinforce heteronormativity and discrimination (9). Evidence from countries such as Kenya, Uganda, and Nigeria shows that nurses commonly rely on personal beliefs and religious values when interacting with LGBTQI patients, resulting in moral judgment and refusal of care (10–12). Furthermore, punitive national laws and widespread social stigma create an environment in which both nurses and patients fear open discussion of gender identity, which hinders accurate assessment and treatment. Similarly, many LGBTQI patients across the region avoid healthcare facilities altogether due to concerns about confidentiality breaches or hostile treatment (13). Consequently, healthcare systems across sub-Saharan Africa often struggle to provide equitable PHC, as professional nurses lack both institutional support and adequate LGBTQI-specific training. For example, Ugandan transgender people report being subjected to invasive and irrelevant questions during routine consultations (14).

In South Africa, despite possessing one of the most progressive constitutional protections globally for sexual and gender minorities, LGBTQI individuals continue to encounter substantial obstacles in PHC settings (15). Professional nurses frequently exhibit uncertainty, discomfort, or judgmental attitudes when handling LGBTQI-related health issues, largely attributable to limited exposure to diversity training during their formal education (14, 16). Furthermore, research indicates that personal beliefs, often rooted in cultural norms and religious values, persistently influence nursing behavior, leading to misgendering of patients, care delays, or dismissive communication (17, 18). On the contrary, LGBTQI patients frequently approach South African health facilities with defensive behaviors, shaped by past experiences of stigma both within and beyond healthcare environments (19). For example, gay and bisexual men have reported being blamed for their sexual health concerns, demonstrating how provider bias continues to undermine patient dignity (20).

Members of the LGBTQI community in the Eastern Cape, South Africa, have reported being ridiculed by nurses when visiting primary healthcare centers within their communities (21). Furthermore, they reported that there is a lack of psychosocial support, gender-minority-friendly STI prevention education, post-exposure prophylaxis, condoms, and lubricants. All these factors contribute to an unwillingness to attend healthcare centers (20), resulting in preventable morbidities and mortalities (22). It was also reported that there has been an alarming increase in HIV infections among LGBTQI youth over the last three decades (23).

Therefore, the study aimed to explore and describe the lived experiences of professional nurses in providing primary healthcare (PHC) services to LGBTQI patients in the Amahlathi Sub-district, Eastern Cape Province, South Africa. The central research question guiding this inquiry was: ‘What are the experiences of professional nurses when providing primary healthcare to LGBTQI patients in Amahlathi Sub-district, Eastern Cape Province?’ The study findings are anticipated to contribute to the development of strategies aimed at strengthening and promoting equitable access to PHC services and improving culturally sensitive care for gender minorities while also mitigating the challenges encountered by professional nurses in-service delivery.

### Theoretical framework

This study was guided by the Health Belief Model (HBM), which elucidates how individuals’ beliefs influence their behaviors (24, 25). The model comprises five core constructs: perceived susceptibility, perceived severity, perceived benefits, perceived barriers, and cues to action (24, 25). In this study, the HBM was not used to explain patient behavior but rather to explore how professional nurses’ beliefs and perceptions influence their provision of primary healthcare (PHC) services to LGBTQI patients in the Amahlathi Sub-district.

1. Perceived susceptibility: Interview probes explored how nurses perceived LGBTQI patients’ vulnerability to specific health conditions (e.g., mental health challenges, substance use). This helped examine whether nurses’ perceptions of risk influenced their clinical attentiveness and screening practices.
2. Perceived severity: Participants were asked to elaborate on the seriousness of health issues affecting LGBTQI patients and the consequences of delayed or inadequate care. This enabled exploration of whether nurses viewed LGBTQI-related health concerns as urgent and deserving of prioritized care.
3. Perceived benefits: Interview questions examined nurses’ views on the value of providing inclusive, respectful, and affirming care. This construct helped determine whether nurses believed that culturally competent practices improve patient trust, disclosure, and health outcomes.
4. Perceived barriers: This construct directly informed the primary question on challenges. Nurses were encouraged to discuss personal, institutional, cultural, and systemic barriers that hinder equitable service provision, including stigma, limited training, and resource constraints.
5. Cues to action: Participants were asked about factors that motivate or enable them to provide inclusive care, such as training, professional guidelines, policy frameworks, or patient encounters. This helped identify structural or professional triggers that influence behavioral change in practice.

## Research material and design

### Research design

A phenomenological qualitative approach was utilized. Through this method, the researchers could explore and describe the comprehensive and in-depth lived experiences of professional nurses who provide PHC services to LGBTQI patients in the Amahlathi Municipality, Eastern Cape Province, South Africa. The professional nurses were asked to share their daily encounters and the challenges they faced while providing primary healthcare services to LGBTQI patients.

### Study setting

The study was carried out in the Amatole district, within the Amahlathi Sub-district in the Eastern Cape, South Africa. The Amahlathi Sub-district is a rural area with an estimated population of 101,286 people who are mostly isiXhosa-speaking. The Amahlathi Sub-district has a total of 51 clinics. The study was conducted in seven selected clinics. Convenience sampling was used to select clinics based on geographical accessibility to the researcher (26).

### Sampling of the participants

A non-probability purposive sampling method was employed to recruit twenty-three (23) professional nurses from seven selected clinics in Amahlathi Sub-district, Eastern Cape, South Africa. The researcher visited the selected clinics to request permission from the Operational Managers to conduct the study in their clinics and to recruit professional nurses. The Operational Managers granted permission to conduct the study and to recruit participants during lunch or after work hours. The researcher individually informed potential candidates about the nature, purpose, and objectives of the study and invited them to voluntarily participate. Those who were willing to participate completed the consent form, and a data collection date was established. All selected professional nurses had experience providing primary healthcare services to LGBTQI patients. Furthermore, they had worked in the selected clinics for more than a year.

### Data collection

Semi-structured interviews with an interview guide were used to collect data; each interview lasted approximately 30–45 min. Fifteen interviews were conducted telephonically when the participants were off duty to avoid disruption of services. The researcher and the participants exchanged telephone numbers after recruitment to set a convenient time for interviews. Eight interviews were conducted in a private room at the selected clinics when the selected participant was eating lunch. The researcher informed the participants that audio recording equipment would be used before the semi-structured interviews began. Participants were guaranteed confidentiality and given the option to withdraw from the study if they felt uncomfortable participating. The following questions were asked to all participants during the interviews; however, other probing questions were dependent on the participant’s response. Probing questions were developed to align with each HBM construct as follows:

1. Perceived susceptibility

How do you perceive the vulnerability of LGBTQI patients to specific health conditions, and how does this influence the way you provide care?

1. Perceived severity

In your view, how serious are the health challenges affecting LGBTQI patients, and how does this shape your clinical response or prioritization of care?

1. Perceived benefits

What do you believe are the benefits of providing inclusive, respectful, and affirming primary healthcare services to LGBTQI patients?

1. Perceived barriers

What personal, cultural, institutional, or systemic challenges do you experience when providing primary healthcare services to LGBTQI patients?

1. Cues to action

What factors, policies, training, or professional influences motivate or enable you to provide inclusive and equitable care to LGBTQI patients?

Data were collected up to the saturation point during the 17 interviews. However, six additional interviews were conducted to confirm data saturation.

### Data analysis

Seven steps of Colaizzi’s descriptive phenomenological method were used to analyze data in order to develop an essential structure of the phenomenon (27). First: using this approach, the researcher and the supervisor read the transcripts of each participant’s interview and listened to the audio-recorded interview several times in order to gain an understanding of the participants and their responses, as well as to familiarize themselves with the data.

Second: every statement in the transcripts that was directly relevant to the research was underlined and listed. Third: meanings relevant to the study that emerged from a careful analysis of the important statements were identified. This necessitated ongoing comparisons between the original transcript, the statements, and the formulated meanings from all study participants. During this step, personal ideas, opinions, beliefs, and assumptions were bracketed, concentrating solely on what the participants had said. Fourth: the identified meanings were grouped into themes shared across all transcripts. The researchers compared the clusters or themes to the original interview and made changes to more accurately represent the participants’ intentions in order to validate them. This necessitated many iterative steps.

Fifth: the researchers then wrote a complete and comprehensive summary of the experiences, including every topic that emerged in step 4. Sixth: the researchers reduced the thorough account to a concise, comprehensive assertion that highlights the key aspects of the professional nurses’ experiences.

Finally, to confirm the reliability of the research findings, available participants were invited to read the entire description to ensure it accurately reflected their experience as a test of the validity of the findings. Only five participants in this study were found to have read the complete, thorough explanation after it was finished.

### Ethical consideration

The ethics clearance certificate was obtained from the Turfloop Research Ethics Committee (TREC) of the University of Limpopo (TREC/582/2022). Permission to access the Amahlathi Sub-district clinics was requested from the Eastern Cape Department of Health Provincial, and permission to conduct the study was obtained (REF: EC_202301_003). The District Manager’s office was contacted by a written letter to request permission, which was granted. The operational nurse managers of the selected clinics permitted the researcher to conduct the study in their clinics. All participants signed consent forms and voluntarily participated in the study. No harm was inflicted upon the participants.

### Trustworthiness

To ensure the research’s trustworthiness, the study employed four criteria: credibility, dependability, confirmability, and transferability. Credibility was established through prolonged engagement with participants and persistent engagement with the interview data. Furthermore, available participants were invited to review the complete description to verify its accurate reflection of their experiences, serving as a test of the findings’ reliability. Dependability was assured by collecting data until saturation was reached. The researchers ensured confirmability by bracketing their thoughts, opinions, and assumptions during data analysis; additionally, field notes and recordings were used to minimize bias. A sample of the interviews was then analyzed by another researcher or a co-coder. Finally, transferability of findings to other contexts was ensured by providing detailed descriptions of the research context, participants, methods, and findings, thereby enabling others to apply the work to their own. This way, the study was thorough, and its findings could be useful in many different settings.

## Results

### Participants’ characteristics

The sample consisted of 16 women and 7 men professional nurses between 25 and 55 years of age. Participants held a Bachelor of Nursing degree or a Diploma in Nursing, with clinical experience ranging from 2 to 30 years. All participants were trained under the R425 nursing program and registered with the South African Nursing Council in General Nursing, Community, Psychiatry, and Midwifery, as illustrated in Table 1.

**Table 1:** Characteristics of the participants.

Participants’ pseudonyms	Gender	Age	Qualification	Year of experience as a PN	Clinic pseudonyms
PN-1	Woman	36	Diploma in Nursing	9	Clinic A
PN-2	Woman	28	Bachelor of Nursing	3	Clinic A
PN-3	Woman	25	Bachelor of Nursing	2	Clinic A
PN-4	Man	25	Bachelor of Nursing	3	Clinic B
PN-5	Woman	26	Bachelor of Nursing	2	Clinic B
PN-6	Woman	32	Bachelor of Nursing	7	Clinic B
PN-7	Man	39	Bachelor of Nursing	15	Clinic C
PN-8	Man	55	Bachelor of Nursing	30	Clinic C
PN-9	Man	42	Bachelor of Nursing	13	Clinic C
PN-10	Woman	29	Bachelor of Nursing	5	Clinic D
PN-11	Man	32	Bachelor of Nursing	7	Clinic D
PN-12	Man	51	Bachelor of Nursing	20	Clinic D
PN-13	Male	46	Bachelor of Nursing	11	Clinic E
PN-14	Man	42	Bachelor of Nursing	14	Clinic E
PN-15	Woman	29	Bachelor of Nursing	5	Clinic E
PN-16	Woman	25	Bachelor of Nursing	4	Clinic F
PN-17	Woman	37	Bachelor of Nursing	3	Clinic F
PN-18	Woman	27	Bachelor of Nursing	3	Clinic F
PN-19	Woman	28	Bachelor of Nursing	3	Clinic G
PN-20	Woman	26	Bachelor of Nursing	5	Clinic G
PN-21	Woman	31	Bachelor of Nursing	4	Clinic G
PN-22	Woman	28	Bachelor of Nursing	4	Clinic G
PN-23	Woman	39	Diploma in Nursing	11	Clinic A

### Interpretation of the findings

Interviews conducted with 23 professional nurses who work in clinics in the Amahlathi area provided researchers with a comprehensive understanding of professional nurses’ experiences in caring for LGBTQI patients. Two main themes and eight subthemes emerged from the data analysis, as presented in Table 2.

**Table 2:** Themes and subthemes.

Theme	Subtheme
1. Challenges of professional nurses related to the provision of PHC to LGBTQI patients	1.1 Professional nurses’ lack of competency regarding LGBTQI issues
	1.2 Professional nurses’ personal experiences, values, and beliefs
	1.3 Professional nurses’ lack of sensitivity toward LGBTQI patients
	1.4 Professional nurses’ confusion regarding LGBTQI issues
	1.5 Inconducive and judgmental attitudes of professional nurses
2. Patient-related challenges to PHC provision to gender minorities	2.1 Patients’ fear of unethical healthcare management
	2.2 Patient reluctance to disclose sensitive information
	2.3 Patients’ defensive behaviors

#### Professional nurse-related challenges to PHC provision to LGBTQI patients

The research revealed that professional nurses in Amahlathi Sub-district clinics encountered multiple obstacles when providing primary healthcare services to LGBTQI patients. Personal experiences, values, beliefs, attitudes, and insensitivity of professional nurses toward LGBTQI patients were found to significantly affect the provision of primary care services to these individuals. Professional nurses also indicated a lack of proficiency in LGBTQI matters, leading to feelings of unease and confusion about LGBTQI health problems. The findings indicate that personal values, beliefs, attitudes, and insensitivity among professional nurses in the Amahlathi Sub-district clinics of the Eastern Cape may negatively influence the quality and equity of primary care services provided to LGBTQI patients. Such influences can result in judgmental behavior, reduced therapeutic communication, and compromised patient–provider relationships.

The following outlines the details of the findings.

##### Lack of competency of professional nurses regarding LGBTQI issues

The study found that professional nurses in the selected clinics are unaware of issues within the LGBTQI community. Professional nurses in the clinics of the Amahlathi Sub-district, Eastern Cape, reported limited knowledge about the sexual practices of LGBTQI individuals, the common health risks associated with their sexual behavior, as well as the use of gender-affirming hormones and their potential side effects. This lack of awareness of LGBTQI issues impacted how they provide health services. For example, when nurses provide health education about sexually transmitted infections, they often focus only on heterosexual aspects rather than homosexual aspects due to their lack of awareness of homosexual behaviors. Participants also highlighted concerns about not knowing what to advise a novice/young LGBTQI person regarding LGBTQI issues and challenges they encounter. Professional nurses expressed concern about their lack of training on gender-minority issues. Professional nurses noted how their lack of training affected their provision of care, which included an inability to provide proper health education and holistic care.

According to the study, professional nurses have varying levels of understanding regarding the healthcare needs of LGBTQI individuals, including their sexual orientations and gender-affirmative services. This gap may affect the inclusive primary health services these patients receive. A lack of competence in comprehending homosexuality may influence how nurses in the Amahlathi region deliver accurate health education or care to homosexual patients.

The Health Belief Model suggests that this gap could influence how professional nurses perceive predisposing factors of illness in their LGBTQI clients, as well as the severity of the illness and its potential consequences. Therefore, this issue will prevent the LGBTQI community from accessing equitable primary healthcare.

As evidenced by:

*“I have been a professional nurse for 14 years, but I don't even know one hormone that is used to transform people's gender. So, if I am going to treat a person with maybe side effects of that particular hormone, how am I going to respond to that because I don't have training in that?” (PN 14)*

*“Young gays and lesbians come in for consultations and guidance, it’s very hard for us to educate them because we ourselves do not know. It is really challenging” (PN 13)*

*“So, I don't know what diseases they may encounter because I don't even know what they do when they have sexual intercourse and stuff. So, I know for myself that the fact is, if it’s a Man and the Woman. I don't know when it is gay, because I don't know how they penetrate themselves.” (PN 12)*

##### Professional nurses’ personal experiences, values & beliefs

Despite a lack of awareness regarding LGBTQI issues, the study also found that professional nurses (PNs) in the Eastern Cape’s Amahlathi Sub-district clinics had difficulties providing care to LGBTQI patients due to their personal backgrounds, values, and beliefs. These backgrounds led them to hold preconceived notions about their patients, creating a barrier to providing care. The participants came from backgrounds that recognized only men and women, requiring them to adjust to these new dynamics.

Additionally, the nurses’ backgrounds, values, negativity, and biases might lead to poor communication and mistrust between professional nurses and LGBTQI patients. As a result, this could lead to prejudice, poor clinical judgments, a lack of cultural awareness, and detrimental health outcomes for LGBTQI individuals. Since these biases could impact the participants’ professional responsibilities, this gap is consistent with the perceived obstacles and triggers to action outlined in the Health Belief Model.

As evidenced by:

*“And sometimes without knowing, you kind of impose because you don't look at the person as they come, you already have your own perception. You have your own values and beliefs taking over before you even hear what the person and the problem they are presenting with” (PN1)*

*'We are Xhosa, we are Africans. According to our belief, men are not supposed to be men either way” (PN9)*

##### Professional nurses lack sensitivity toward LGBTQI patients

The professional nurses in the clinics of the Eastern Cape Amahlathi Sub-district admitted to not being sensitive to the issues of gender minorities. This included not knowing how to refer to gender minority patients and, at times, unintentionally saying offensive things. They attributed this to a lack of training or sensitization regarding gender minority issues. This, in turn, results in LGBTQI patients feeling judged, uncomfortable consulting, or experiencing fears.

The relationship and trust between LGBTQI patients and the professional nurses at Amahlathi Sub-district clinics may be harmed by this lack of awareness. This conclusion will also impact how people view their vulnerability, obstacles, and signals to action in accordance with the Health Belief Model. Using disrespectful words and failing to provide adequate care to patients can make them feel undervalued or disrespected. As a result, during consultation, LGBTQI patients may have difficulty being forthright and honest, which will affect the treatment they receive.

The lack of sensitivity toward LGBTQI patients may weaken the connection and trust between the patients and the professional nurses at Amahlathi Sub-district clinics. This is perceived as a barrier because it will impact how professional nurses view LGBTQI patients’ vulnerability, obstacles, and signals to action, in accordance with the Health Belief Model. The use of disrespectful language and failing to provide adequate care to LGBTQI patients can make them feel undervalued or disrespected. As a result, during consultation, LGBTQI patients may find it hard to communicate openly and honestly, which will affect the treatment they receive.

As evidenced by:

*“The gay community is never comfortable being consulted by nurses because they feel that nurses would not be nice or saying something that would be offensive. But not intentionally.” (PN 2)*

*“The LGBTQI issues are not something all healthcare workers are necessarily sensitized to. We are not all sensitized to their specific needs, and I think it is due to a lack of training.” (PN 11)*

*“I work in the antenatal clinic; I had a lesbian patient. Obviously, I did not understand how you got pregnant. Another nurse asked her. The patient responded and said that she did not appreciate the question.” (PN 15)*

##### Confidence and discomfort of nurses about LGBTQI issues

The findings revealed that professional nurses in the Eastern Cape Amahlathi Sub-district clinics exhibited judgmental attitudes toward LGBTQI+ patients. This behavior may stem from a lack of awareness or personal background, values, or beliefs. These nurses were observed making judgmental remarks, contributing to an unsupportive clinic environment for LGBTQI+ patients. The professional nurses reported that their judgmental attitudes negatively impacted follow-up sessions, leading to more complicated cases. The study also indicated that older nurses were more likely to hold preconceived values, beliefs, and backgrounds compared to younger nurses. This difference might be attributed to younger nurses reporting greater familiarity with the LGBTQI+ community. However, most participants acknowledged that judgmental responses sometimes occur automatically, even if unintended, due to unfamiliarity with LGBTQI+ issues, resulting in unintentional imposition and judgment.

Nurses’ discomfort regarding this patient group is perceived as a barrier to equitable and quality primary healthcare services, as described by the Health Belief Model. This barrier can impede proper health assessments, diagnoses, and treatments, leading to a lower standard of care. As evidenced by:

*“Consultations are very complicated. You do not know whether to call the patient Ms. or Mr. And if you ask them what I should call you, they will be offended.” (PN 4)*

*“You must find the right choice of words to treat them and avoid offending, you know. So, you don't know what to say and what is wrong for you to say and what is right for you to say. So, for me, it makes me feel a bit confused or maybe not sure how to respond as a nurse.” (PN 1)*

*“Our Clinic has male and female toilets. You don't even know which bathroom to refer them to too when you want specimens.” (PN 12).*

*“I had a gay come in for itchy vulva, no anal canal. Upon evaluating it, I realized it was so open. I asked him what was going on, after a very long interrogation he admitted that it was due to a sex position. That consultation was the most uncomfortable for me. I could see he was uncomfortable too” (PN8)*

##### Inconducive and judgmental attitudes

As previously discussed, this study found that professional nurses in the Eastern Cape’s Amahlathi Sub-district clinics were inadequately trained or sensitized to LGBTQI+-related health issues. This deficiency resulted in a lack of competence in providing LGBTQI+ care, further exacerbated by their personal beliefs, values, and experiences. This study observed that these factors contributed to clinics becoming unsupportive and judgmental environments. Participants reported that judgmental remarks created an unconducive atmosphere for both professional nurses and patients.

The unsupportive and unwelcoming clinic environment can also influence perceived susceptibility, barriers, and cues to action, according to the Health Belief Model. Such an environment may diminish LGBTQI patients’ trust in healthcare professionals and make them reluctant to disclose crucial health information, ultimately affecting their health outcomes. This is evidenced by:

*“It's a normal reaction of a human to judge a man dressed as a woman or vice versa. So, when they walk in, you already have preconceived ideas about them.” (PN17)*

*“Sometimes we as nurses are rude to them, we shout at them, so due to their previous encounters, they will just become angry.” (PN 8)*

*“When they are at the clinics, they are judged a lot, especially by the older generation. They are judged by a lot of people.” (PN 14)*

#### Patient-related challenges affecting PHC provision to gender minorities

Although this research revealed that professional nurses in the clinics of the Eastern Cape’s Amahlathi Sub-district experienced challenges related to their own actions and backgrounds, they also noted that other challenges stemmed from patient behaviors. These included patients’ reluctance to disclose sensitive information, patients’ fear of unethical healthcare management, and patients’ defensive behavior.

##### Patients’ fear of unethical healthcare management

The healthcare professionals highlighted that LGBTQI patients appear hesitant to access healthcare due to anticipated judgment, discrimination, and breaches of confidentiality. According to professional nurses, these patients lack information and fear unethical behavior or mismanagement that may arise from professional nurses if they disclose their sexuality. This included breaches of confidentiality and judgment. At times, this fear stemmed from previous experiences of gender minorities.

Fear of unethical health management in this study might be caused by mistrust in nurses’ ability to maintain professional confidentiality. These findings also align with the Health Belief Model, as patients’ fear of unethical healthcare management serves as a barrier. Furthermore, it will affect how nurses perceive the seriousness of LGBTQI patients’ diseases and the actions to be taken, resulting in difficulty providing suitable and personalized care in the Amahlathi Clinics. As evidenced by:

*“I think the reason that perpetuates these people not to come in for examinations and physical examinations is because, you know, as people will say. So, I think they have the fear that maybe I will say something to the people that live in the community and such things can destroy somebody's self-esteem and whatever they hold in the community.” (PN 3)*

*“When they access the healthcare system, they are afraid of harassment… They are afraid of discrimination, and they could also be afraid of poor-quality treatment if they reveal their identity.” (PN 23)*

*“They have faced judgment in the past, even from healthcare workers. Now it is not easy for them to understand the system has been revamped. There is a new generation of healthcare providers who serve everyone equally.” (PN 15)*

##### Patients’ reluctance to disclose sensitive information

Due to fear of unethical behavior and judgment, professional nurses noted that LGBTQI patients delayed seeking help in clinics, and even when they did, they were not completely honest about their sexuality and sexual behaviors. According to the professional nurses in the clinics of the Eastern Cape Amahlathi Sub-district, this caused challenges in history taking, diagnosis, and treatment. The nursing professionals stated that this concealment of sexuality affected the quality of care they provided to gender minorities.

The Health Belief Model in this study is used to guide the findings, particularly focusing on barriers encountered by nurses, the perception of condition seriousness, and cues to action. The behavior of these nurses contributes to LGBTQI patients avoiding or delaying seeking healthcare, leading to missed opportunities for early detection, timely interventions, and the worsening of conditions that could otherwise be managed or prevented. As evidenced by:

*“They would come with bloody stools, and they would not mention that they have this problem because I'm the way I am sexually, which is how I got it.” (PN1)*

*“They take long to come to healthcare and even if they do come, they are not entirely honest about their sexuality, making it a bit complicated when it comes to diagnosis. It affects even the history taking, delaying us from helping the patient.” (PN2)*

##### Patients’ defensive behavior

According to professional nurses, LGBTQI patients often present to clinics with a defensive demeanor, which is reportedly attributable to their previous healthcare experiences. Participants reported that LGBTQI patients exhibit a negative attitude and defensive behavior, possibly due to prior negative treatment. This impacts the professional nurses’ ability to gather history as their questions are met with unsettling remarks. This results in difficulties in providing care, as patients from gender minorities are offended by questions and respond negatively.

The patient’s defensive behavior may suggest a communication barrier between nurses and LGBTQI patients at Amahlathi Sub-district clinics. This poses a significant challenge in gathering complete patient information, making it difficult to formulate an accurate diagnosis, resulting in unaddressed health needs and poor outcomes. As evidenced by:

*“Most of the time they come with an attitude because they know that mainly in the healthcare system, they are being treated unfairly just because of the sexuality they chose. Yeah So, they will just come with attitude and that it is something very difficult to deal with because you will try to be nice. The person already has those angers.” (PN 8)*

*“I think they've been terrorized enough. They don't understand when we want knowledge or judging. I remember one of fellow student had asked a question and then that gay didn't respond very well to the question. Because I feel like sometimes, we ask only to obtain knowledge and not judge because we want to learn to live with them and even protect them from other people.” (PN 13)*

In summary, the findings of this study integrate with the three key components of the Health Belief Model, namely perceived susceptibility, perceived barriers, and cues to action. The findings showed that the perceptions of professional nurses and a lack of competency regarding homosexuality impacted the delivery of primary healthcare services to LGBTQI patients in Amahlathi Sub-district clinics. This leads to nurses exhibiting negative attitudes, judgmental behavior, discomfort, and a lack of sensitivity when treating LGBTQI patients. Consequently, LGBTQI patients reported reluctance to visit the clinic, share accurate health information, and trust healthcare workers or services.

## Discussion

The findings of this study demonstrate that professional nurses in the clinics of the Eastern Cape Amahlathi Sub-district still lack adequate competence to provide comprehensive primary care to LGBTQI patients. This aligns with long-standing evidence that healthcare workers worldwide exhibit limited knowledge about LGBTQI sexual practices, gender-affirming hormone therapies, and specific health risks (9, 28). This knowledge gap is often linked to insufficient coverage of LGBTQI health in nursing and medical curricula (9). Similarly, a South African study found that healthcare workers frequently reported feeling unprepared or poorly equipped to treat patients from sexual and gender minorities (15). In our findings, nurses’ inability to provide accurate sexual health education or appropriate advice to LGBTQI youth mirrors the gaps highlighted in the international literature, reinforcing that knowledge deficiencies remain a critical barrier to equitable healthcare.

The results also illuminate how the personal beliefs, cultural values, and moral frameworks of nurses shaped their interactions with LGBTQI patients in the Amahlathi Sub-district clinics. Cultural and religious beliefs of healthcare workers were found to often influence perceptions of sexual and gender minorities, resulting in implicit or explicit bias (29). Similarly, African contexts demonstrate increased challenges due to conservative socio-cultural norms (11). Participants in this study referenced their cultural background (e.g., Xhosa traditions) as contributing to the difficulty in accepting gender diversity, consistent with one study that reported South African nurses often internalize social stigma, which impacts professional behavior (30). In contrast, research shows that when healthcare providers undergo exposure, training, or reflection on their biases, attitudes can improve (31). This suggests that while cultural beliefs can hinder care, targeted interventions have the potential to shift attitudes positively (32).

Institutional and systemic barriers further compounded inequalities in LGBTQI healthcare. Nurses in this study cited a lack of guidelines, a lack of sensitization programs, and uncertainty regarding clinic procedures (e.g., pronouns and bathroom use). Structural factors such as unprepared facilities, limited protocols, and the absence of inclusive policies contribute to alienating health environments for LGBTQI patients (33). Similarly, Fuller (34), argues that discriminatory health systems amplify fear, prevent help-seeking, and reinforce power imbalances. These findings are also supported by one of the studies that emphasize that without institutional leadership and policy clarity, even well-intentioned providers struggle to implement inclusive care (35). Therefore, the discomfort and confusion expressed by nurses in this study are symptoms of deeper systemic gaps rather than individual shortcomings alone.

Finally, professional nurses in the Eastern Cape’s Amahlathi Sub-district clinics reported various challenges related to LGBTQI patients, such as fear, non-disclosure, and defensive behaviors, indicative of the effects of historical and ongoing discrimination within healthcare settings. According to nurses, fear of mistreatment or breaches of confidentiality contributes to these behaviors, a view that is supported by evidence demonstrating that LGBTQI individuals frequently avoid accessing healthcare due to anticipated stigma (36–38). Similarly, non-disclosure of sexual practices leading to delayed diagnosis corresponds to the minority stress model, which explains that concealment is a coping mechanism developed in response to discrimination (39). This reluctance can lead to missed diagnoses and inappropriate treatment, particularly in sexual and reproductive health (40). According to a previous study, nurses agreed that the defensive behaviors of LGBTQI patients are caused by the previous negative experiences with healthcare providers, which create mistrust (41). Therefore, the interactions observed in this study are part of a cycle in which provider discomfort and patient fear mutually reinforce barriers to care.

The application of the Health Belief Model (HBM) enriched the interpretation of findings by moving beyond a descriptive account of challenges toward an analysis of the belief structures underpinning nurses’ practices. While previous studies have identified stigma, lack of training, and personal beliefs as barriers to LGBTQI-inclusive care, the HBM allowed these findings to be interpreted within a structured cognitive framework. For example, negative attitudes and discomfort were understood as perceived barriers, limited prioritization of LGBTQI health concerns reflected variations in perceived severity, and recognition of mental health risks aligned with perceived susceptibility. Furthermore, the absence of institutional training emerged as a weak cue to action, explaining why inclusive practices were inconsistently implemented. Thus, HBM did not merely echo established insights but provided a theoretical lens that clarified how individual beliefs interact with structural constraints to shape clinical behavior.

### Study recommendations

Based on the study findings, it is recommended that comprehensive LGBTQI-focused training be integrated into both pre-service and in-service nursing education to improve clinical competence and reduce discomfort when treating gender-diverse patients. Clinics in the Eastern Cape’s Amahlathi Sub-district should implement clear institutional guidelines, including protocols for respectful communication, the use of pronouns, and appropriate access to facilities, to create inclusive environments. Sensitization programs should be prioritized to address personal biases, cultural beliefs, and judgmental attitudes that hinder equitable care. Strengthening confidentiality policies and reinforcing ethical conduct can help rebuild trust and reduce patients’ fear of discrimination. In addition, clinics must provide targeted health education materials that address the sexual and reproductive health needs of LGBTQI individuals. Collaboration with LGBTQI community organizations is recommended to support culturally safe service delivery, while ongoing monitoring and evaluation should be used to ensure sustained improvements in practice.

### Strengths and limitations of the study

A key strength of this study is its use of rich, in-depth qualitative interviews, which allowed professional nurses to openly share their experiences, attitudes, and challenges in providing primary care to LGBTQI patients, thus generating nuanced insights that are often overlooked in quantitative research. The study also contributes to a limited body of South African literature focused specifically on rural or semi-rural contexts such as Amahlathi, offering context-specific evidence that can inform local policy and training. Additionally, the inclusion of nurses with varying years of experience provided a diverse range of perspectives. However, the study is limited by its reliance on self-reported data, which may be influenced by social desirability bias, particularly regarding attitudes toward LGBTQI individuals. The findings are also drawn from a single municipality, which can restrict the generalizability of the results to other regions. Furthermore, the absence of the perspectives of LGBTQI patients limits the ability to fully understand both sides of the healthcare interaction.

## Conclusion

This study highlights significant challenges facing professional nurses in providing primary healthcare services to LGBTQI patients in the Amahlathi Sub-district, Eastern Cape, revealing gaps in knowledge, cultural sensitivity, and institutional support. Nurses’ limited competency, influenced by inadequate training and deeply rooted personal and cultural beliefs, contributed to discomfort, confusion, and judgmental attitudes that negatively shaped patient experiences. At the same time, professional nurses reported that the fear of discrimination among LGBTQI patients, the reluctance to disclose sensitive information, and defensive behaviors further complicate clinical interactions, creating a cycle that undermines effective care delivery. According to nurses, these challenges highlight the urgent need for comprehensive LGBTQI-focused education, sensitization programs, and clear institutional guidelines to strengthen inclusive and ethical healthcare practices. Addressing these structural and interpersonal barriers is essential to improve access, quality, and equity of care for gender and sexual minorities within the South African primary healthcare system.

## Data Availability

The original contributions presented in the study are included in the article/supplementary material, further inquiries can be directed to the corresponding author.
